# Supplementary material for: Synthesis of cationic liposome nanoparticles using a thin film dispersed hydration and extrusion method
Source: PLoS One. 2024 Apr 9;19(4):e0300467. doi: 10.1371/journal.pone.0300467 (PMC11003666; doi:10.1371/journal.pone.0300467)
Supplement: S1 File — Protocol published on protocols.io regarding Synthesis of cationic liposome nanoparticles using a thin film dispersed hydration and extrusion method. (PDF) [file pone.0300467.s001.pdf]

Liposome Synthesis Protocol : Synthesis of cationic liposome nanoparticles using a thin film dispersed hydration and extrusion method. V.2

Version 1 is forked from Liposome Synthesis Protocol : Synthesis of anionic and cationic unilamellar liposome nanoparticles using a thin film dispersed hydration and extrusion method.

Joanna Carroll[1], Noah Daly[1], Julie Rose Mae Mondala[1], Alancasey[2], Brijeshtiwari[3], James F Curtin[4], Alessandro Cazzolla[1]

[1]School of Food Science & Environmental Health, Technological University Dublin, Dublin, Ireland;
[2]School of Physics, Clinical and Optometric Sciences, Technological University Dublin, Dublin, Ireland;
[3]Teagasc Food Reserch Centre, Ashtown, Dublin, Ireland ;
[4]Faculty of Engineering & Built Environment, Technological University Dublin, Dublin, Ireland

Cancer Therapeutic Lab - TU Dublin

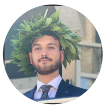

Alessandro Cazzolla

VERSION 2

JUL 13, 2023

OPEN ACCESS

DOI:
dx.doi.org/10.17504/protocols.io.dm6gpjyx1gzp/v2

Protocol Citation: Joanna Carroll, Noah Daly, Julie Rose Mae Mondala, Alancasey, Brijeshtiwari, James F Curtin, Alessandro Cazzolla 2023. Liposome Synthesis Protocol : Synthesis of cationic liposome nanoparticles using a thin film dispersed hydration and extrusion method.. **protocols.io** https://dx.doi.org/10.17504/protocols.io.dm6gpjyx1gzp/v2
Version created by Alessandro Cazzolla

License: This is an open access protocol distributed under the terms of the Creative Commons Attribution License, which permits unrestricted use, distribution, and reproduction in any medium, provided the original author and source are credited

ABSTRACT

Nanoparticles are the future of targeted and sustained drug delivery, yielding significant opportunities in addressing and treat challenging diseases such as cancer (Grenader et al., 2012; Luminari et al., 2010; Shoji et al., 2014). These nano vesicles can be developed into intelligent systems which can encapsulate medicinal and imaging agents, while still possessing stealth properties. Manipulating the size, surface properties, and material composition of these novel systems can help deliver drugs to specific tissues and provide controlled release therapy. Among them, lipid nanoparticles, particularly liposomes, have been identified as promising and adaptable drug vesicles. When compared to standard drug delivery systems, they have superior qualities such as site-targeting, prolonged or controlled release, drug protection from degradation and clearance, superior therapeutic efficacy, and lower toxic side effects.
Liposomes are phospholipid vesicles that form approximately spherical bilayer shapes. These spherical vesicles can contain unilamellar or multilamellar internal concentric lipid bilayers trapping aqueous space. Due to this structure, liposomal vesicles allow for a wide variety of drugs with lipophilic and hydrophilic properties to be encapsulated simultaneously with hydrophobic/lipophilic molecules being inserted into the bilayer membrane and hydrophilic molecules being entrapped in the aqueous compartments. Beginning with Gabizon et al., 1982 and Tardi et al.,

Protocol status: Working
We use this protocol and it's working

Created: Mar 13, 2023

Last Modified: Jul 13, 2023

PROTOCOL integer ID: 78686

Keywords: Liposome, Thin film dispersed hydration method, anionic, cationic, DOTAP, DSPC

1985, liposome researchers have developed a wide range of liposomal drug carriers, by primarily employing small-scale laboratory techniques. The size of liposomes has a significant role in the effective delivery of anticancer drugs to the tumour site. Liposome size has been demonstrated to affect its time in blood circulation, tumour accumulation, tumour retention and the drug release. According to data so far, a liposome diameter of below 200 nm is the ideal size to achieve efficient drug delivery especially when targeting the brain and crossing the blood brain barrier (Gao et al., 2013; Kulkarni & Feng, 2013).
This protocol aims to outline the steps involved in the synthesis of cationic and anionic liposomes with an approximate uniform size of 90 nm for targeted drug delivery in tumour cells. This method employed the use of thin-film dispersed hydration one of the most popular methods for preparing liposomes.

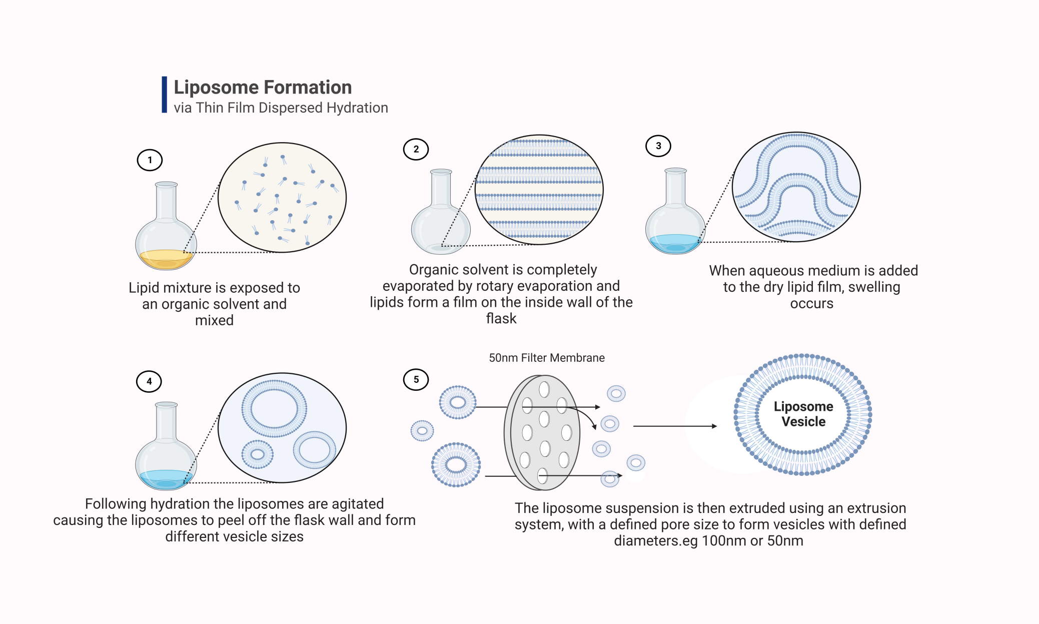

Figure 1. One of the most popular methods for preparing liposomes is the thin-film approach. It is based on the development of a thin lipid film that forms on the inner wall of the rotary evaporator flask. The film is then later hydrated with an aqueous solution coupled with the vigorous shaking, allowing the film to peel off the flask and form liposomes. The liposome rich solution is then subjected to extrusion to obtain a homogeneous mixture with defined diameters. The biggest plus of this method is its high reproducibility even when working with small quantities of compound.
(Image created using BioRender, 2023)

GUIDELINES

- If the liposome solution is not turbid after re-hydration and mixing, synthesis has possibly failed.
- Ensure to vortex the liposome solution before extrusion and before analysis. This helps to avoid extra aggregations that may occur.
- Extruder systems should be assembled according to the guidelines laid out by the manufacturer of the liposome extruder used. Improper assembly can lead to loss of sample. If during extrusion the passes are difficult to complete, change filter supports and polycarbonate membrane and continue.
- After extrusion and analysis, store the liposomes at 4°C. This step is important as acid and base hydrolysis can occur in lipids with ester-linked hydrocarbon chains. Temperature has a significant impact on the rate of hydrolysis; hence lipid suspensions should be stored in the refrigerator.

MATERIALS

Chemicals

- 12-dioleyol-3-trimethylammonium-propane (DOTAP) **Merck Catalog #890890P-200MG**
- Cholesterol **Merck Catalog #C8667-1G**
- Chloroform 99+% **Fisher Scientific Catalog #10236110**
- Ultrapure Water

Encapsulating compounds

- Propidium Iodide **Merck Catalog #81845-100MG**
- Ursolic Acid **Merck Catalog #U6753-100MG**
- Fluorescein isothiocyanate isomer I **Merck Catalog #F7250-250MG**

Equipment

- Round bottom flask (50ml)
- Retort stand
- Droppers
- Hot and stirring plate
- Magnetic stirrer
- Rotary evaporator
- Vortex
- Avanti Micro Extruder Set **Merck Catalog #610000-1EA**
- Polycarbonate Membrane 50nm **Merck Catalog #610003-1EA**

SAFETY WARNINGS

- Laboratory coat, glasses and gloves must be worn at all times.
- Chloroform is a neurotoxic chemical and all work with it must be carried out inside a chemical fume hood.
- The Safety Data Sheets for each chemical used should be reviewed, and risk assessment of the activity conducted prior to using any chemicals.
- In the case where extrusion at high T_c is required, it is recommended to use heat resistant gloves to avoid burns as the extrusion system can become very hot to touch.

BEFORE START INSTRUCTIONS

- Ensure that the transition temperature of the chosen lipid is known and work above this.

Synthesis of liposomes via thin film dispersed hydration

1 *The thin-film dispersed hydration method can be used to prepare anionic and cationic liposomes and encapsulated liposomes with (fluorescent dyes, bio compounds or drugs).*
In a round bottom pyrex flask (50 ml), add [M] 7 millimolar (mM) of the chosen lipid and [M] 3 millimolar (mM) of cholesterol and dissolve in 5 mL of chloroform. (if the aim is to encapsulate lipid-soluble drugs, dissolve the lipid-soluble drug during this step. Eg. Ursolic Acid 1:20 (w/w).

2 Stir the solution with a magnetic stirring rod for 00:15:00 just above the lipid transition temperature (Tc). 15m

3 Once stirring is complete, remove the stirring rod from the flask and let the chloroform solvent evaporate using a rotary evaporator at 40 °C.

Note
Evaporation of the solvent should be done at a slow consistent rate until all solvent removed and flask is dry.

4 Further dry the flask by incubating it overnight in an oven set at just above the T_c of the chosen lipid.

5 Re-hydrate the lipids that have formed a thin film around the inside of the flask by adding to

2-3 mL mL of ultrapure water. The volume of water added to the flask depends on the desired final concentration. (If aiming to encapsulate a water-soluble drug or reporter chemical, dissolve the compound during this step. Eg. Propidium Iodide, or FITC 1:20 (w/w).

6 Stir the rehydrated solution for 00:30:00 , using a magnetic stirring rod, making note again to stir the solution on a hot plate with a temperature just above the lipid Tc. 1h

Note

The solution must be turbid when stirring is complete. If after 00:30:00 of stirring the solution is not turbid, it may be necessary to manual manoeuvre the stirring of the solution. This will aid in the removal of the lipids from the side of the flask.

7 Once solution is finished stirring, remove the stirring rod and vortex the solution for 00:02:00 . 2m

8 After vortexing, store the solution at 4 °C overnight. This is to prevent acid and base hydrolysis reactions taking place in lipids with ester-linked hydrocarbon chains. Temperature has a significant impact on the rate of reaction so the solution should be refrigerated to slow this process.

Method for Extrusion of Liposomes

9

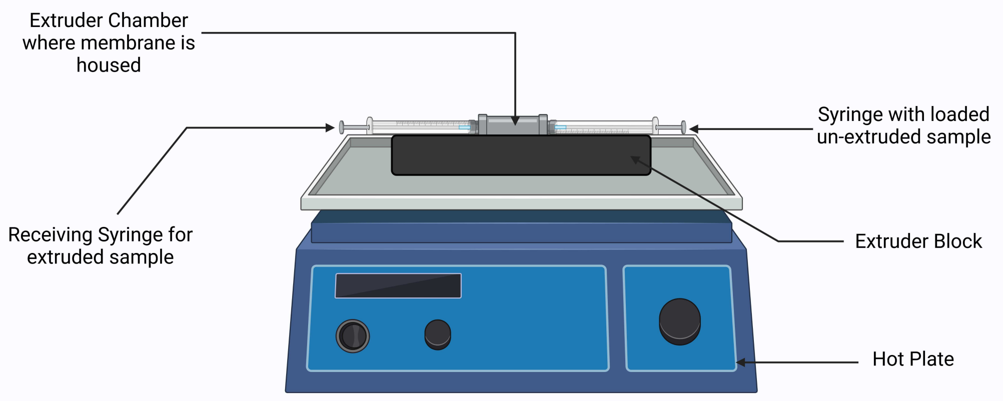

Figure 2. Example display of a Miniextruder system, for liposomes with high Transition temperatures, extrusion should be carried out on a hot plate. **(Image created using BioRender, 2023)**

Place a 100nm polycarbonate membrane into the extruder chamber. For this protocol, we have

used an Avanti mini extruder system *(see steps on how to do this on the Avanti website: https://avantilipids.com/divisions/equipment-products/mini-extruder-assembly-instructions).* The system allows large vesicles to be reduced to unilamellar vesicles by extrusion through defined pore membrane sizes in an efficient manner. The protocol can be adapted for other mini extruder systems which should be used in accordance with the manufacturer's guidelines.

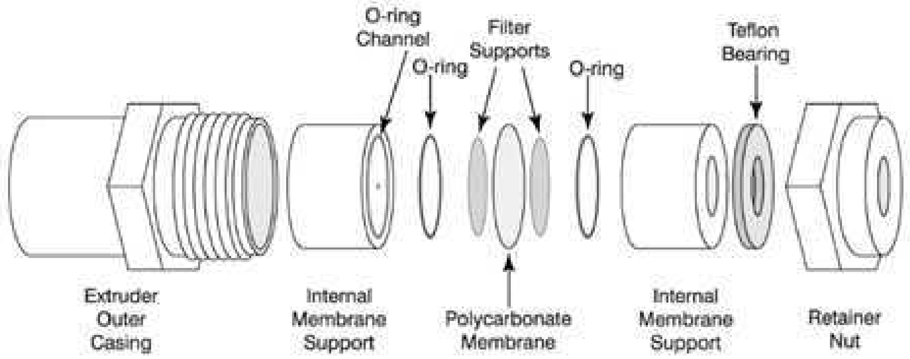

Figure 3. Extruder chamber set up and components where the polycarbonate membrane and filter supports are housed**.** The quantity of passes through the membrane determines the particle size distribution of liposome vesicles produced by extrusion system. (https://avantilipids.com/divisions/equipment-products)

10 Once chamber is sealed closed, position the extruder chamber into the extruder block (See **Figure 2**).

11 With the syringe, draw up 1 mL of the freshly vortexed liposome solution.

12 Position the syringes into the extruder block as seen in (See **Figure 2**) and passage the liposome solution through the 100nm polycarbonate membrane 7 times. Be sure that the syringes are fully inside the block.

13 Once the liposome solution has been brought down to 100nm, open the extruder chamber and replace the 100nm membrane with a 50nm polycarbonate membrane.

14 Rinse the system with ultrapure water prior to the 50nm extrusion. Once flushed, repeat the extrusion process.

15 Store extruded liposomes in 4 °C to maintain liposome stability.

Characterization of Synthesized Liposomes

16 *Liposome properties, such as particle size, poly dispersity index (PDI) and zeta potential (ZP) are important parameters to study when synthesising liposomes for drug delivery. Dynamic Light Scattering (DLS) is a commonly used equipment for determining the particle size, PDI and ZP of liposome solutions. Studying the morphology of synthesised liposomes is also a helpful characterization step and be done using Scanning electron microscope (SEM).*

DLS - Determines the average particle size of the liposomes and the polydispersity of the vesicle size.
Zeta Potential – Determines the average surface charge on the liposome vesicles.
Scanning electron microscope (SEM) – Used to observe the liposomes morphology.
HPLC – Used to analyse the separation and quantification of the lipid components in the liposome formulations.
NMR - Can be used to investigate if the liposomes are unilamellar or multilamellar as they provide different NMR resolution.
