# Supplementary material for: Synthesis of cationic liposome nanoparticles using a thin film dispersed hydration and extrusion method
Source: PLoS One. 2024 Apr 9;19(4):e0300467. doi: 10.1371/journal.pone.0300467 (PMC11003666; doi:10.1371/journal.pone.0300467)
Supplement: Attachment — Submitted filename: Response to Reviewers.docx [file pone.0300467.s002.docx]

Monday 28^th^ September 2023

**RE: PLOS ONE Decision: Revision required [PONE-D-23-22881]**

Dear Dr Kumar,

Many thanks for reviewing our manuscript entitled: *Synthesis of cationic liposome nanoparticles using a thin film dispersed hydration and extrusion method*. We now enclose an updated manuscript where we have addressed all of the reviewer feedback, and we also enclose an accompanying rebuttal letter responding to each point raised.

We have also removed the funding from the acknowledgements section. Please include the following as our amended funding statement: This study was funded by a TU Dublin Research Scholarship (AC) [www.tudublin.ie](http://www.tudublin.ie) and by Science Foundation Ireland, 21/FFP-A/9189 (JC) [www.sfi.ie](http://www.sfi.ie).

With best wishes,

James and Alessandro ([james.curtin@tudublin.ie](mailto:james.curtin@tudublin.ie) and [alessandro.cazzolla@tudublin.ie](mailto:alessandro.cazzolla@tudublin.ie))

**Journal Requirements:**

1. Please ensure that your manuscript meets PLOS ONE's style requirements, including those for file naming. The PLOS ONE style templates can be found at

https://journals.plos.org/plosone/s/file?id=wjVg/PLOSOne_formatting_sample_main_body.pdf and

https://journals.plos.org/plosone/s/file?id=ba62/PLOSOne_formatting_sample_title_authors_affiliations.pdf

We can confirm that the manuscript meets PLoS One style requirements.

2. Thank you for stating the following in the Acknowledgments Section of your manuscript:

"This study was funded by a TU Dublin Research Scholarship and Science Foundation Ireland, 22/FFP-A/9189. The authors wish to thank TU Dublin, ESHI, and FOCAS Research Institutes for the use of facilities and the support of technical staff and research support office staff"

We note that you have provided funding information that is not currently declared in your Funding Statement. However, funding information should not appear in the Acknowledgments section or other areas of your manuscript. We will only publish funding information present in the Funding Statement section of the online submission form.

Please remove any funding-related text from the manuscript and let us know how you would like to update your Funding Statement. Currently, your Funding Statement reads as follows:

"TU Dublin Research Scholarship 2022 (AC) www.tudublin.ie

Science Foundation Ireland, 22/FFP-A/9189 (JC) www.sfi.ie

The funders did not and will not have a role in study design, data collection and analysis, decision to publish, or preparation of the manuscript."

Please include your amended statements within your cover letter; we will change the online submission form on your behalf.

We have amended the acknowledgments and removed reference to funding agencies in this section. Please include the following as our amended funding statement: *This study was funded by a TU Dublin Research Scholarship (AC) www.tudublin.ie and by Science Foundation Ireland, 21/FFP-A/9189 (JC)* [*www.sfi.ie*](http://www.sfi.ie)*.*

3. We note that you have stated that you will provide repository information for your data at acceptance. Should your manuscript be accepted for publication, we will hold it until you provide the relevant accession numbers or DOIs necessary to access your data. If you wish to make changes to your Data Availability statement, please describe these changes in your cover letter and we will update your Data Availability statement to reflect the information you provide.

We are making no changes to our Data Availability Statement and will finalise this and release the DOI number once the manuscript is approved.

4. Your ethics statement should only appear in the Methods section of your manuscript. If your ethics statement is written in any section besides the Methods, please move it to the Methods section and delete it from any other section. Please ensure that your ethics statement is included in your manuscript, as the ethics statement entered into the online submission form will not be published alongside your manuscript.

The paragraph on ethics statement has been moved to the methodology section, lines 111-120

5. Please include a separate caption for each figure in your manuscript.

This is now done, the list of figure and table captions has been added in the manuscript.

6. Please ensure that you refer to Figure 7 in your text as, if accepted, production will need this reference to link the reader to the figure.

This has now been corrected.

7. Please include captions for your Supporting Information files at the end of your manuscript, and update any in-text citations to match accordingly. Please see our Supporting Information guidelines for more information: http://journals.plos.org/plosone/s/supporting-information.

This is now done, the list of supporting information files with captions has been added at the end of the manuscript.

8. We note you have not yet provided a protocols.io PDF version of your protocol and/or a protocols.io DOI. When you submit your revision, please provide a PDF version of your protocol as generated by protocols.io (the file will have the protocols.io logo in the upper right corner of the first page) as a Supporting Information file. The filename should be S1_file.pdf, and you should enter “S1 File” into the Description field. Any additional protocols should be numbered S2, S3, and so on. Please also follow the instructions for Supporting Information captions [https://journals.plos.org/plosone/s/supporting-information#loc-captions]. The title in the caption should read: “Step-by-step protocol, also available on protocols.io.”

The pdf related to the protocol was changed to S1_file as specified by the guideline provided, see line 110

Please assign your protocol a protocols.io DOI, if you have not already done so, and include the following line in the Materials and Methods section of your manuscript: “The protocol described in this peer-reviewed article is published on protocols.io (https://dx.doi.org/10.17504/protocols.io.[...]) and is included for printing purposes as S1 File.” You should also supply the DOI in the Protocols.io DOI field of the submission form when you submit your revision.

The DOI for the protocol is included in the manuscript.

If you have not yet uploaded your protocol to protocols.io, you are invited to use the platform’s protocol entry service [https://www.protocols.io/we-enter-protocols] for doing so, at no charge. Through this service, the team at protocols.io will enter your protocol for you and format it in a way that takes advantage of the platform’s features. When submitting your protocol to the protocol entry service please include the customer code PLOS2022 in the Note field and indicate that your protocol is associated with a PLOS ONE Lab Protocol Submission. You should also include the title and manuscript number of your PLOS ONE submission.

The protocol is uploaded in protocols.io.

**Reviewer #1**:

26-27 An optimised method was developed ...
What is the optimised of your method? Can you add parameters you have optimised? You are using combination of 100 nm and 50 nm membrane, specific concentration of liposomes, etc.

As suggested, we explained better the method, also giving quantities that were used in the synthesis, see page 2, lines 29-34

37-38 Lipid nanoparticles, particularly liposomes, have been identified as potentially viable and flexible drug vesicles ...
This sounds to me like referring to a new potential material. Liposomes are 60 years old and nearly 30 years already on the market. I think this should be reflected in the introduction.
72-74 According to available evidence, a liposome diameter of less than 200 nm is the optimal size for effective drug administration, particularly when targeting the brain and crossing the blood brain barrier (13)
Here, you are citing your older version of this protocol?
Generally I have problem with the introduction that it states general knowledge about liposomes that is known for decades and in the same time citing very recent articles that are not primary sources of these informations. From your introduction, liposomes seem to me like a super new potential material that yet needs to be studied.

Emphasis regarding the discovery of nanoparticles that occurred about 60 years ago, see page 2 and 3, lines 43-45. The correct article has been cited, lines 87

99-100 dissolved in a fixed amount of chloroform and stirred for 15 minutes above the lipid’s transition temperature, Tc.
What amount of chloroform and at which temperature? In the protocol, the chloroform amount is stated, temperature not. Were these quantities optimised as it is stated in abstract?
As suggested the quantities and stirring temperature used were specified in the text, lines 128-133

119 ... and surface charge.
I would be very careful with connections of Zeta potential and surface charge. This may vary with ionic strength, size (as you even show)

All sentences where the term surface charge was used for the zeta potential have been modified as suggested.

126 - please check italic/normal characters in the equation
Nowhere in the methods I see the procedure for the doxorubicin encapsulation and purification. If there was purification used, the calculation of EE does not make sence as there was doxorubicin removed from the solution
Equation corrected as suggested. Specified the return to starting volume before measuring absorbance regarding EE of doxorubicin, lines 197-200

190 - liposomi
Fixed

193-194 Here and on many other places you overusing the digits in calculation. There is no point is showing that your measured value is 64.087 ± 33.98. Reasonable amount of digits need to be shown in the whole publication.
Values regarding size and PdI were changed using 1 decimal place and 2 decimal places, respectively

3.2 Extrusion study passes: The study you are here performing was surely done many times and even the manual from the manufacturer contains this information, for example here: https://www.ncnr.nist.gov/userlab/pdf/E134extruder.pdf
The method reported is a modification of the one proposed by Avanti, lines 235-237

212-214 ... on the other hand, values less than ±30 can result in particle aggregation, flocculation, and precipitation due to the van der Waal forces of attraction which result in physical instability of the colloidal suspension (15).
Here, you are citing the paper in which this information is stated also with citation. It is very popular to give some border for the Zeta potential but from my point of view this is not correct. The colloidal stability is given by DLVO theory by the combination of electrostatic repulsion and van der Walls attraction. If this value exists it is material specific (in this case for liposomes).

A change was made regarding what the zeta potential represents for us in terms of stability, keeping in mind that there would be other forces to take into account, see page 11, lines 235-237

218 - 219 ... confirming the effectiveness of the proposed extrusion method ...
Here it seems like a new method.
The proposed method has been changed to the amended method, line 257.

3.5 Encapsulation % If I understand correctly, you are using Ultracentrifugation and then without the knowledge about volumes, you are subtracting the concentrations. I think this is not correct approach.
Specified the return to starting volume before measuring absorbance regarding EE of doxorubicin

The final part about cytotoxicity is not in the protocol. If something has added value considering the previously published protocols it is probably this part.
In the results of cytotoxicity test, the ones with longer time from synthesis is more toxic for the cells. This probably means that the doxorubicin leaked from the liposomes and killing the cells, but still you are stating that "Storage at 4 ºC appears to be the best, with stability maintained over 16 weeks." (line 312).
Specified how stability tests refer to DOTAP and DOTAP-PI and not doxorubicin.

Figure 1 is 1:1 copy from Functionalized liposomes for targeted breast cancer drug delivery, Nel et al. Bioactive Materials 2023 and it is not adding any information. I would suggest either cite the source or to add some relevant info.

The image was created by taking cues from the reported article, the correct citations will be made in the caption of the figure.

**Reviewer #2:**

1. The language throughout the manuscript causes significant misunderstandings and more importantly misstatements. It needs more care than this journal requires.
   Changes have been made to avoid misunderstandings
2. There are several sentences in the manuscript where the punctation marks are missing thus making it really hard to follow.
   a. Line 190, the word liposomes was misspelled
   b. Lines 233-234, what does the authors mean by intensity zeta size and number zeta size?
   c. The sentence of lines 241-242 needs to be modified.
   d. Lines 270-272, “Results in terms of size will be discussed considering the number and not the intensity, to show better the actual increase, if present, of the liposomes size.” needs some simplification.
   e. Line 274, what does no encapsulated case mean and in line 277 the author described it as unencapsulated. Please maintain consistency across the manuscript.
   f. Line 276, the text in the manuscript needs to be written in past tense.

A better explanation has been provided regarding the paragraph dealing with size in terms of distribution by intensity and number see lines 277-284. Line 241-242 has been modified, see line 323. Lines 270-272 has been modified, see line 291-292. All "no encapsulated" has been changed to "unencapsulated"

3. In Figure 4B, the term Zeta Size in the graph needs to change to Size.
4. The text in the manuscript says Figures 5A and 5B, whereas in the actual figures, ther is no A and B in Figure 5.
5. The text says Figures 6A, 6B and 6C, whereas the actual figures are 7A, 7B and 7C.

Figure modified and figure citations fixed

6. There are several manuscipts describing the synthesis of cationic liposomes, what makes this one unique. The novelty needs to be discussed further in detail.

The novelty of the proposed method has been better emphasized, with added commentary on the cell lines used, lines 96-98

7. Use of cationic lipids comes with its own challenges and toxicities. The potential toxicities involved need to be mentioned in the introduction.

In accordance with what has been suggested the toxicity of using cationic lipids has been specified in the introduction, lines 59-61.

8. Furthermore, the stability of the synthesized liposomes are attributed to the highly positive zeta potential. Longer shelf-life does not make the liposome safe. A safety study such as a hemolysis study needs to be performed to evaluate the toxicities on cell membranes.
10. The authors need to discuss more about the drop in zeta potential from time zero to 1 week old samples. The zeta potential at time zero was 47.90 mV whereas in 1 week old samples stored in cold were 7.90 mV and 18.30 mv for DOTAP and DOTAP-PI respectively.
15. Furthermore, the authors need to study the safety of these cationic liposomes in healthy cells in addition to the cancer cells.

Subsequent studies will need to be done regarding the decrease in zeta potential from week 0 to week 1, lines 309-311. Improved explanation provided regarding cytotoxicity measured as weeks go by, with subsequent studies that may also be done on normal cell lines, see lines 368-374

9. Authors also need to perform drug release study as a part of stability study as well.

Stability test was made on DOTAP and DOTAP-PI no doxorubicin
11. The justification of such high standard errors in the zeta potential needs to be discussed as well.

We amended in the manuscript. See lines 212-213 and 254-255
12. A justification of why Doxorubicin was used to study the encapsulation % needs to be described in the manuscript and not Propidium Iodide.

Reason for use of doxorubicin was specified in the introduction, lines 87-91
13. Cytotoxicity need to be performed with freshly prepared liposomes as well.

Emphasis regarding cytotoxicity performed on the three cell lines, specifying that they were performed with freshly made liposomes, see line 354.

14. The authors need to clearly discuss why the bigger size impacted the cytotoxicity.
Improved explanation provided regarding cytotoxicity measured as weeks go by, with subsequent studies that may also be done on normal cell lines, see lines 368-374
