# Supplementary material for: Synthesis of cationic liposome nanoparticles using a thin film dispersed hydration and extrusion method
Source: PLoS One. 2024 Apr 9;19(4):e0300467. doi: 10.1371/journal.pone.0300467 (PMC11003666; doi:10.1371/journal.pone.0300467)
Supplement: Attachment — Submitted filename: Response to Reviewers.docx [file pone.0300467.s003.docx]

Response to Reviewer 1

I appreciate the minor changes you have performed in order to improve the manuscript, such as better phrasing, clarification about Zeta potential, number of digits, reference in figure 1.

We thank the reviewer for the agreeing that the changes made improved the manuscript. We will now address the other issues that have been since raised below.

Still I have following major issues that were already stated in first response:
1) Introduction still looks like the liposomes are really new and potential material even after you added 2 lines to the text. Prime example 90-91: "These negative consequences have propelled researchers to create advanced, alternative drug delivery systems that use nanotechnology to transport DOXO (15)"
Here, it again is cited very recent article on DOXO-iron particles which is really something new in development. But the liposomes with DOXO are totally not new. There are dozens of already marketed DOXO - liposome products in market. <https://go.drugbank.com/drugs/DB00997>. First of them nearly 30 years FDA approved.
I would really suggest to perform deep literature review to have a more relevant introduction.

We thank the reviewer for the suggestions. We have extensively rewritten the introduction, highlighting the origins of liposome research, recent advances in drug delivery systems including clinically approved examples of this, and setting the context for this method to accelerate drug development and delivery systems in research labs worldwide. These changes can be seen in the manuscript on lines 43-48, lines 92 to 98 and lines 103 to 105.

2) There are at least 2 protocols at protocol.io that are dealing with the same problem. Both of them are better than the one here reported.
<https://www.protocols.io/view/two-step-protocol-preparation-and-extrusion-of-pho-n2bvj95xlk5w/v3>
<https://www.protocols.io/view/liposome-encapsulation-of-hydrophilic-and-hydropho-rm7vzymorlx1/v1>

We thank the reviewer for highlighting these two protocols. One method was developed previously by our laboratory, but since then we have improved it, for example we have adjusted the quantities of cholesterol and lipids to make the extrusion more straightforward, and we can confirm that our new method reported in this manuscript is a more robust method for drug delivery studies.  The other method is a two step protocol for preparing and extruding liposomes. The method differs from ours in a number of ways including the liposome composition. The authors do not include cholesterol. We have found that cholesterol is important for liposome stability and biocompatibility reasons. Moreover, the temperature used by these authors in their protocol is always 60 °C. This is higher than the temperatures we typically use. However, we have not directly compared this method with ours so we are unable to say which one is better.

I would suggest to add the part with 2D cell culture and cell viability assay in order to form complete protocol which can help students and researchers to do basic tests in liposomes.

Thank you very much for the suggestion. In the PLoS Lab Protocol, we included a step by step protocol for the synthesis of cationic liposomes in protocols.io. We provided additional experiments and results to provide context in the accompanying PLoS Lab Protocol manuscript. Regarding 2D cell culture and cell viability assays, we believe that these experiments fall into that second category, contextualising the protocol with applications and expected results, rather than being a core part of the actual synthesis protocol.

3) There is still not stated how the Doxorubicin was encapsulated. There are numerous methods for this, the most common ones use salt gradients: for example review here: 10.3390/pharmaceutics14020254.
A protocol for this method would be beneficial. Furthermore, the equation for EE calculation is adding concentrations as an extensive value. This is not correct. Using ultracentrifugation, the volumes of all fractions need to be known. There is a plenty of research done on the EE experimental evaluation: <https://doi.org/10.1016/j.ijpharm.2017.11.035>. Your reported EE around 85 % corresponds to the EE with using salt gradients but it is nowhere stated.

Thank you very much for the suggestion about doxorubicin encapsulation. We have provided this information in the methods section (please see lines 148 and 149).

Thank you very much for the comment on EE. We have clarified the method (please see line 212-215). The EE changed from 84.8% to 81%, and this is been updated in line 36 of the Abstract, Table 1, line 367 and line 398 and 399 of the Expected Results and Discussion section.

4) 96-98 There is now statement about the novelty of the work. I seriously doubt this statement as the liposomes were intensively tested through last 50 years (for example here: Kitamura et al., 1996, <https://aacrjournals.org/cancerres/article/56/17/3986/502500/Intrathecal-Chemotherapy-with-1-3-Bis-2>) but if this publication should serve as a protocol it is not necessary to have original research.

We thank the reviewer for the comment. We have clarified this statement about the work carried out in glioblastoma cell lines (please see line 110-111).
